# The Effect of Honey Supplementation on Skeletal Muscle‐Related Inflammatory Markers Among Military Graduates After Overtraining

**DOI:** 10.1002/hsr2.70428

**Published:** 2025-02-13

**Authors:** Esmail Karami, Mohammad Reza Parvizi, Mohammad Reza Izadi, Emad Jowhari Shirazi

**Affiliations:** ^1^ Cognitive Neuroscience Research Center, AJA University of Medical Sciences Tehran Iran; ^2^ Exercise Physiology Research Center, Life Style Institute, Baqiyatallah, University of Medical Sciences Tehran Iran; ^3^ Physiology Department AJA University of Medical Sciences Tehran Iran; ^4^ Department of Physical Education and Sports Sciences Imam Hossein Comprehensive University Tehran Iran; ^5^ General Practitioner, Student Research Committee AJA University of Medical Sciences Tehran Iran

**Keywords:** honey supplementation, inflammatory markers, military graduates, overtraining

## Abstract

**Aims and Objectives:**

Honey possesses specific anti‐inflammatory properties. We evaluated the effects of oral honey supplementation on skeletal muscle‐related inflammatory markers among military graduates after overtraining.

**Methods:**

This randomized double‐blind controlled trial was conducted on 42 overtrained military graduates who were randomly assigned to receive either honey supplementation (5 mL of 12% honey solution twice daily for 6 weeks) or a placebo for 6 weeks. In our study, we utilized Milk Vetch Sahand honey as the intervention. Serum levels of C‐reactive protein (CRP), tumor necrosis factor‐α (TNF‐α), aldolase A, and creatine kinase (CK) were evaluated using the enzyme‐linked immunosorbent assay method before and after the intervention.

**Results:**

A total of 21 male participants with a mean age of 20.75 years were included in each group. No significant differences were found between the groups in the pre‐intervention assessment of inflammatory biomarkers. However, in the post‐intervention assessment, participants who received honey exhibited significantly smaller increases in CRP (*p* < 0.001), TNF‐α (*p* = 0.001), aldolase A (*p* < 0.001), and CK levels (*p* < 0.001) compared to the controls. The mean changes in these biomarkers were notably lower in the honey group indicating a potential anti‐inflammatory effect of honey.

**Conclusion:**

This study demonstrates that honey supplementation significantly reduces inflammatory biomarkers such as TNF‐α, CRP, aldolase A, and CK in military graduates experiencing overtraining, underscoring its potential to alleviate inflammation associated with intense physical training. To confirm and extend these findings, further research with larger sample sizes and longer follow‐up periods is recommended.

## 1 Introduction

Skeletal muscles represent the most prevalent tissues in the human body, with a pivotal role in protein and energy metabolism, movement, and stability. Muscle injuries from strains, lacerations, contusions, ischemia, burns, or rigorous exercise training might interfere with these processes [1]. Unusual exercise, particularly eccentric [2] muscular contractions during military training may cause muscle injury that manifests as ultrastructural changes in the muscle tissue, efflux of myocellular proteins, and symptoms like muscle soreness and swelling, diminished muscle strength, and limited range of motion [2]. Muscle injuries may adversely affect daily activities, quality of life, and occupational capabilities [3].

The primary goal of basic military training programs is to develop conscripts' physical fitness, strength, and endurance in a short period so that they can perform combat‐specific tasks. Imbalanced high‐intensity exercise training and recovery can lead to overreaching and overtraining, a short‐term drop in performance that can take several days or weeks to recover from. A prolonged imbalance, in turn, may result in overtraining syndrome, characterized by a long‐term decrease in performance despite continuous training, with recovery taking months or years [3].

Expression of inflammatory biomarkers can promote both muscle growth and loss. Intramuscular inflammatory signaling is essential for mediating the regenerative response to muscle fiber damage [4, 5]. Kazeem A. et al. reported the changes in acute phase proteins following moderate and extended exercise training [6]. They discovered that high‐intensity exercise training raised plasma C‐reactive protein [7] levels, which may have a role in the etiology of several disorders, such as metabolic syndrome, diabetes, and cardiovascular disease [8]. Therefore, finding a new therapeutic way to decrease the expression of inflammatory biomarkers after high‐intensity military exercise training may minimize injury‐related muscle contraction impairments. Numerous studies have reported the effectiveness of physiotherapeutic, pharmaceutical, and dietary approaches for easing the symptoms of exercise‐induced muscle injury, with varying degrees of success [2].

Honeybees produce honey by collecting nectar from various plants, resulting in a diverse range of honey types, each with unique flavors, colors, and nutritional profiles. The macro‐ and micro‐nutrient composition of honey is influenced by several factors, including the specific bee species, the types of flowers visited, and the environmental and processing conditions. For instance, clover honey is known for its light color and mild flavor, while buckwheat honey is darker and has a robust taste. In total, honey contains over 200 different chemicals, including sugars, proteins, enzymes, minerals, vitamins, amino acids, and polyphenols [9, 10]. Honey is an immune‐modulating substance with a dual function [1]: anti‐inflammatory activities via downregulation of inflammatory transcription factors (NF‐B and MAPK) and/or suppression of the production of pro‐inflammatory cytokines and [2] stimulation of the production of inflammatory mediators, such as prostaglandin E2 (PGE2) and cyclooxygenase‐2 (COX‐2) [11].

Milk Vetch Sahand honey, a rare and highly valued variety, is particularly notable for its rich nutritional content and numerous health benefits. Derived from the nectar of Milk Vetch flowers, this honey has a characteristic yellow color and is recognized for its anti‐inflammatory and antioxidant effects, which boost the immune system and scavenge free radicals. Milk Vetch Sahand honey continues to be appreciated in both traditional and modern healthcare contexts. In a study by Nakajima et al. in 2013 investigating the effects of three types of Japanese honey, including Chinese milk vetch honey, on wound healing in mice, it was observed that these honeys initially reduced wound area during the inflammatory phase. Combining these insights, the role of Milk Vetch Sahand honey as an immune‐modulating agent, particularly in the context of inflammatory responses, is a promising area for further exploration, especially in applications such as supporting skeletal muscle recovery after intense physical training [12, 13].

According to the narrative review, in patients with cancer, neuro‐inflammatory disorders, and liver injury honey has been reported to inhibit the effects of pro‐inflammatory factors such as TNF‐α and IL‐6. Five papers in systematic review on the effect of honey on exercise performance also showed improvements in bone health after honey consumption in combination with jumping exercises or aerobic dance [14, 15]. Amanollahi et al. showed that high‐intensity interval training programs along with honey consumption could have favorable effects on inflammatory markers in 38 healthy young men with a sedentary lifestyle [16]. Evidence suggests the role of cytokines in overtraining syndrome in animal experiments and highlights the need to conduct human studies to confirm this link [17]. However, little is known about the advantages of honey supplementation as an adjunct to mandatory military training. This study aims to evaluate the effects of oral honey supplementation on the level of skeletal muscle‐related inflammatory markers including serum CRP, tumor necrosis factor‐α (TNF‐α), aldolase A, and creatine kinase (CK) among military graduates after overtraining.

## 2 Methods

### 2.1 Ethics Statement

In accordance with the Declaration of Helsinki, this study received ethical approval from the Ethics Committee of the Sport Sciences Research Institute of Iran (IR. SSRC. REC‐2211‐1933); all participants provided written informed consent for inclusion before participation in the study.

### 2.2 Study Design

This prospective, double‐blind clinical trial was conducted at the AJA University of Medical Sciences, Tehran, Iran, from February 2023 to May 2023. The inflammatory parameters of military graduates receiving honey after overtraining (treatment group) were compared with cases receiving a placebo (control group). Participants included male military graduates from the Officer Training School, aged 20–30, belonging to a specialized military unit with comparable anthropometric profiles. They had undergone extensive military training and voluntarily agreed to participate in the study. Additionally, it is worth mentioning that during the study period, all participants consumed the same standard Persian cuisine for every serving. This diet reflected the meals they had been routinely consuming for years, with no changes to the menu or dietary patterns.

The exclusion criteria were any underlying diseases, disabilities, or traumatic injuries, use of immunosuppressive drugs, use of nonsteroidal anti‐inflammatory drugs within 15 days before the intervention, history of malignancy, primary and secondary hemostasis disorders, and decision to withdraw from the study. We also excluded participants with incomplete data sheets.

### 2.3 Confirmation of Overtraining

The main symptoms of overtraining were assessed using a questionnaire from the French Sports Medicine Association to validate both the psychological and physiological aspects of overtraining. This questionnaire consists of 54 psychoanalytical questions with yes or no answers. The participants who answer yes to more than 20 questions are considered to have early symptoms of overtraining [18, 19, 20]. The validity and reliability of this questionnaire were calculated and reported to be 0.95 in a similar study in Iran, suggesting it to be a suitable measure to assess overtraining [21].

The ratio of cortisol to testosterone was used to confirm overtraining objectively [22]. Overtraining is diagnosed if this ratio decreases by more than 30% and this reduction continues in the long term [22]. Studies indicated that the overtraining questionnaire scores directly correlate with cortisol levels and inversely correlate with testosterone levels and the testosterone‐to‐cortisol ratio [23, 24].

### 2.4 Grouping

**Figure 1:**
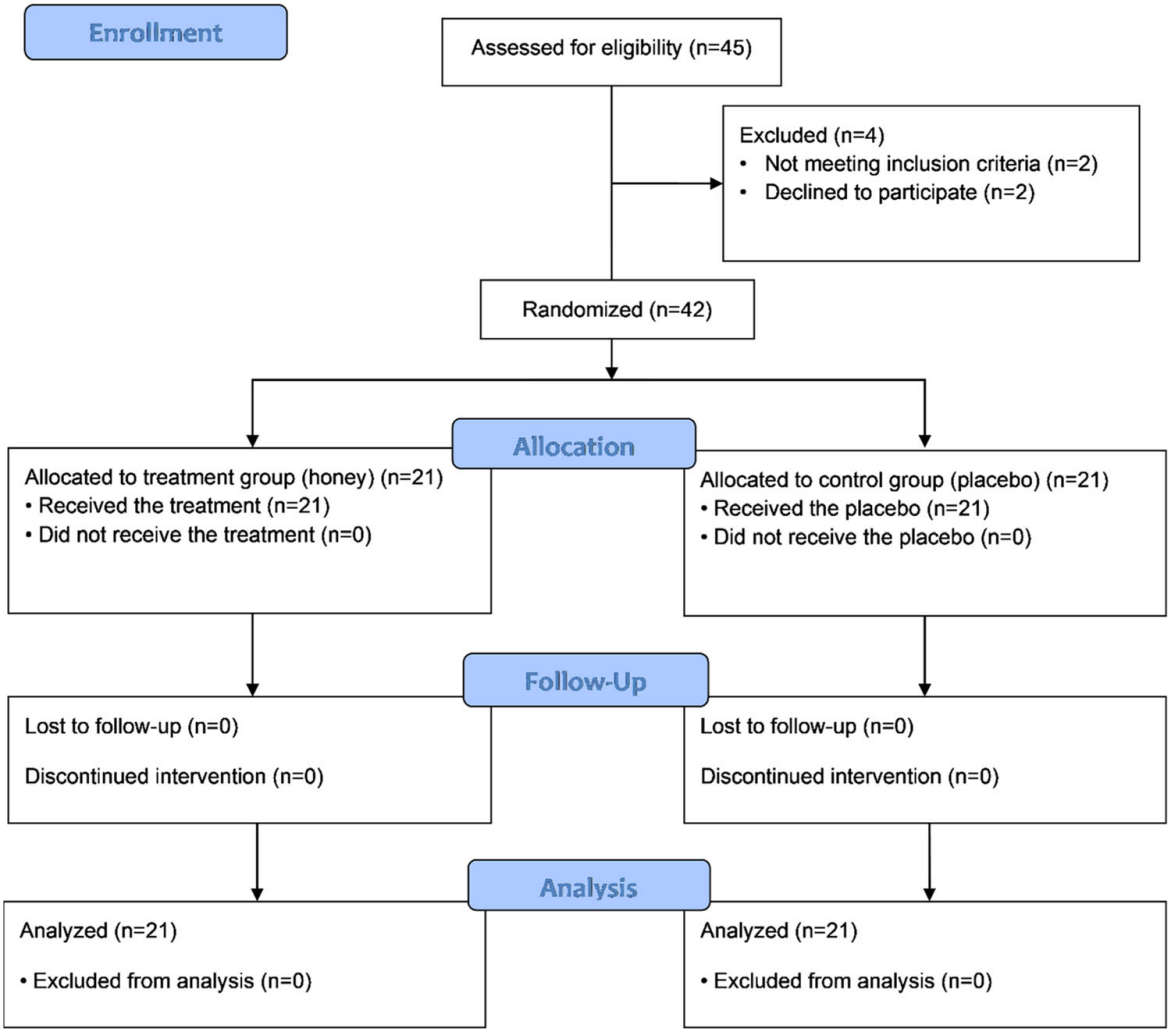
CONSORT flowchart of the study.

The CONSORT flowchart of the study is shown in Figure 1. Forty‐six overtrained subjects were assessed for eligibility by specialists based on the inclusion and exclusion criteria. Forty‐two eligible participants were randomly allocated into the two groups (21 in the treatment group and 21 in the control group) using a block randomization procedure, with subjects matched in each block based on age.

### 2.5 Intervention

Before the start of the intervention, a 1‐month washout period was implemented. During this time, the soldiers remained in the barracks and engaged in light physical exercises, while training classes were held. Following this washout phase, participants were randomized into the honey supplementation or placebo groups. In the intervention group 5 mL of 12% honey oral solution was given for each kilogram of body weight twice daily at 8:00 a.m. and 4:00 p.m. for 6 weeks. The placebo group was given the same amount of oral solution containing one and a half tablets of Canderel (aspartame; a sweetener without calories, KRUGER, Germany) at the same hours for the same duration. We used Milk Vetch Sahand honey for this study [25]; Milk vetch Sahand honey is derived from the nectar of the milk vetch flowers, known for its rich nutritional profile. The total phenolic content was notably high indicating strong antioxidant properties. The pH was slightly acidic, and the electrical conductivity was low, suggesting lower ionic content [25]. We have summarized the composition of the product in Table 1.

**Table 1:** Composition of Milk Vetch Sahand honey used in the intervention group.

Parameter	Details
Honey type	Milk Vetch Sahand honey
Color	Yellow, resembling typical honey color
pH	4.08 ± 0.4
Water content	15.59% ± 0.74%
Total phenolic content	777.58 ± 469.72 mg GAE/100 g
The absorbance (ABS450)	129.26 ± 82.4 mAU
Electrical Conductivity	0.38 ± 0.09 mS/cm

Both the participants and the examiner were blinded to the intervention solution and one of the authors directly observed the entire consumption process to ensure that the supplementation was taken as prescribed and completed fully.

Intervening with the physical activity program of the subjects was impossible due to the importance, variety, and need for training according to military missions. Hence, the physical activity program of the subjects was according to the military's routine and was the same for all our participants, usually endurance‐type in the form of long‐distance running, military exercises, and combat preparation training.

### 2.6 Assessments

To determine the levels of aldolase A, CK, CRP, TNF‐α, cortisol, and testosterone, 5 mL of blood was sampled from the brachial vein of the subjects at the same time of the day before and 6 weeks after the intervention. The sample was poured into plastic tubes containing serum‐separating gel and lacking anticoagulants. After centrifuging samples at 3000 rpm for 15 min, the separated serum was kept at −70°C until all samples were ready for laboratory analysis.

The levels of the above‐mentioned factors were determined using the enzyme‐linked immunosorbent assay (ELISA) method with the relevant kits. The serum TNF‐α levels were determined according to the manufacturer's instructions using a commercial human TNF‐ELISA Kit (Quantikine, R&D Systems, and Minneapolis, MN, USA). The intra‐ and inter‐assay variation coefficients were 5.1% (*n* = 20) and 6.1% (*n* = 20), respectively. The serum CRP levels were measured via the nephelometric method (Immage 800 Beckman Coulter).

Participants' generalized conditions were monitored through weekly check‐ups, where vital signs (including blood pressure, heart rate, and temperature) were recorded. Participants were also asked about any adverse effects, changes in appetite, energy levels, and overall health status using a questionnaire.

### 2.7 Statistical Analysis

Data were statistically analyzed using the SPSS version 22 program (SPSS Inc. Chicago, IL, USA). Subjects with missing data were excluded. Normality was assessed using the Kolmogorov‐Smirnov test. An independent samples *t*‐test was used to compare the intervention and placebo groups, and paired *t*‐tests were employed to compare pre‐and post‐intervention values. Two‐tailed *p* values of below 0.05 denoted statistical significance.

## 3 Results

The present study comprises a total of 42 male participants with a mean age of 20.75 ± 1.75 (SD) years and a mean BMI of 19.08 ± 4.68(SD) kg/m^2^ who were randomly allocated into two groups. The mean pre‐intervention testosterone level was 5.5 ± 4.50 (SD) pg/mL and the mean cortisol level was 19.66 ± 5.91 (SD) ng/mL. Notably, the post‐intervention ratio of cortisol to testosterone decreased by 34% in both groups confirming the overtraining effect. According to the findings, the two study groups exhibited no significant variation in TNF‐α at the baseline (13.32 ± 2.66 vs. 13.06 ± 5.51, *p* = 0.84). After the intervention, a significant increase in TNF‐α was observed in both groups with a within‐group *p* value of less than 0.001 in both groups. However, the postintervention level of TNF‐α was significantly lower in participants who received honey supplementation than in the control group (Intergroup *p* = 0.004) as shown in Table 2. Additionally, the mean change in the placebo group was approximately two times higher than the intervention group (Table 2, Intergroup *p* = 0.001).

**Table 2:** Serum levels of inflammatory markers (Mean ± SD) in the intervention (honey supplementation) and placebo groups (*n* = 21).

Variables	Groups	Honey Supplementation	Placebo	*p* value* (Intergroup)
TNF‐α (pg/mL)	Pre‐intervention	13.32 ± 2.66	13.06 ± 5.51	0.84
	Post‐intervention	18.07 ± 2.89	21.27 ± 3.80	0.004
*p* value** (within‐group)		< 0.001	< 0.001	
Changes (Mean ± SD)		4.74 ± 1.61	8.20 ± 4.19	0.001
CRP (mg/dL)	Pre‐intervention	4.33 ± 2.57	4.42 ± 3.20	0.91
	Post‐intervention	8.66 ± 4.52	11.66 ± 4.46	0.03
*p* value** (within‐group)		< 0.001	< 0.001	
Changes (Mean ± SD)		4.33 ± 2.53	7.23 ± 2.30	< 0.001
Aldolase A (U/L)	Pre‐intervention	5.33 ± 0.98	5.14 ± 0.84	0.50
	Post‐intervention	7.45 ± 1.03	8.37 ± 0.79	0.002
*p* value** (within‐group)		< 0.001	< 0.001	
Changes (Mean ± SD)		2.11 ± 0.74	3.22 ± 0.98	< 0.001
Creatine Kinase (U/L)	Pre‐intervention	77.47 ± 31.78	76.00 ± 41.59	0.89
	Post‐6intervention	205.90 ± 59.10	265.14 ± 89.00	0.01
*p* value** (within‐group)		< 0.001	< 0.001	
Changes (Mean ± SD)		128.42 ± 34.42	189.14 ± 63.07	< 0.001

Abbreviations: CRP, C‐reactive protein; TNF‐α, tumor necrosis factor‐α.

* Intergroup comparison (Intervention vs. Placebo) was done using an independent sample *T*‐test.

** Within‐group comparison was done using a paired sample *T*‐test.

The serum CRP levels were comparable between the two groups during the pre‐intervention stage, as evidenced by a *p value* of 0.91. A significant increase in CRP levels was observed in both groups (8.66 ± 4.52 vs. 11.66 ± 4.46, within‐group *p* < 0.001). This rise was significantly more prominent in the controls compared with the intervention group (4.33 ± 2.53 vs. 7.23 ± 2.30, intergroup *p* = 0.001).

We observed no statistically significant disparity in aldolase A levels between the two groups during the pre‐intervention phase (*p* = 0.50). Nevertheless, postintervention, the group receiving honey supplementation exhibited a significantly smaller increase compared to the control group (7.45 ± 1.03 vs. 8.37 ± 0.79, postintervention intergroup *p* = 0.002). Despite the significant increase in both arms (*p* < 0.001), the increment was notably more pronounced in the control group in contrast to the treatment group (2.11 ± 0.74 vs. 3.22 ± 0.98, *p* < 0.001).

**Figure 2:**
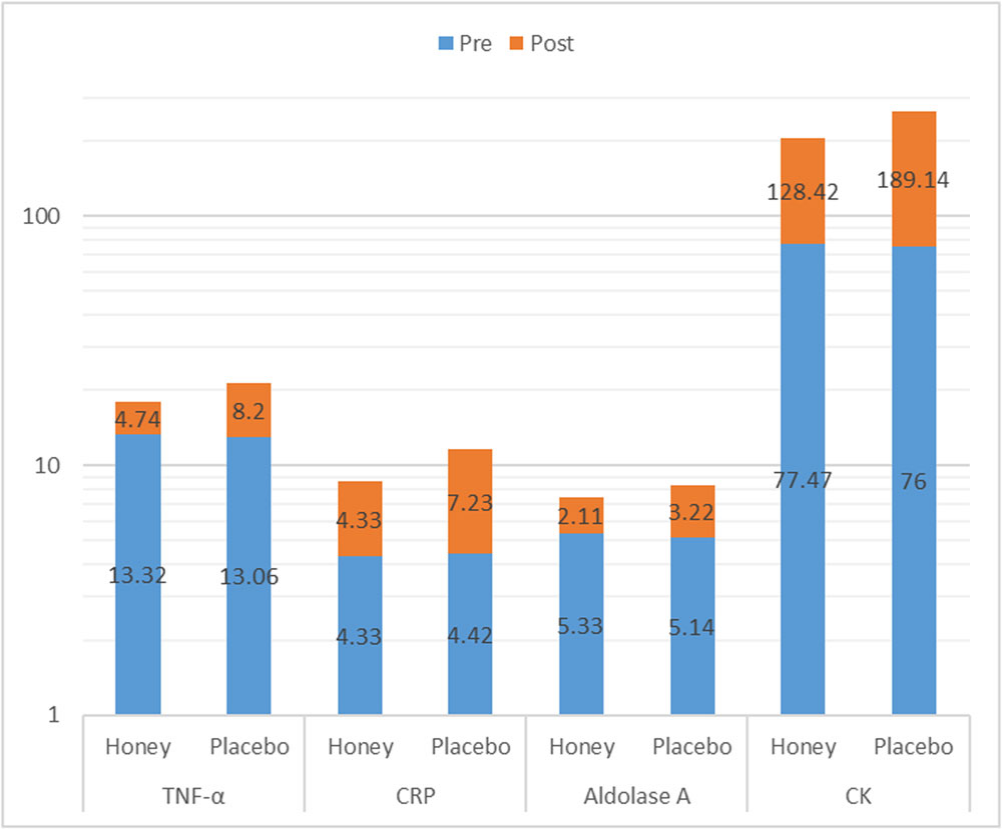
Clustered column chart comparing baseline (pre) and posthoney supplementation (post) mean serum levels of inflammatory markers include TNF‐α (Tumor Necrosis Factor‐alpha), CRP (C‐reactive protein), aldolase A, and CK (creatine kinase) in the honey and placebo groups. Blue bars represent the baseline values, while orange bars indicate the magnitude of change after the intervention. The honey group shows significantly smaller changes in inflammatory marker levels compared to the placebo group, highlighting the potential anti‐inflammatory effects of honey supplementation.

The pre‐intervention levels of CK in both arms were comparable (*p* = 0.89). Both groups experienced a significant rise in CK levels (within‐group *p* < 0.001). However, the treatment group demonstrated lower levels of CK following the intervention (205.90 ± 59.10 vs. 265.14 ± 89.00, *p* = 0.01) (*p* < 0.001). The increase was significantly more pronounced in the placebo group compared with the treatment group (189.14 ± 63.07 vs. 128.42 ± 34.42 *p* < 0.001). Figure 2 is visualizing the comparison of baseline and post‐honey supplementation mean levels of inflammatory markers in the honey and placebo groups.

Additionally, participants' overall generalized conditions were monitored during the 6‐week treatment period. Vital signs remained within normal ranges, no adverse effects were reported, and there were no significant changes in their general health status, including appetite and energy levels.

## 4 Discussion

To the best of our knowledge, the present study is the first clinical trial to investigate the anti‐inflammatory effect of honey supplementation in overtrained individuals by measuring the serum levels of four inflammatory biomarkers including CRP, TNF‐α, aldolase A, and CK. The results indicate significant changes of these biomarkers in both study arms, more pronounced in controls, and support the anti‐inflammatory role of honey in this context.

The adverse effects of overtraining syndrome on muscular performance highlight the importance of taking preventive measures such as appropriate training plans and dietary supplementation [26, 27]. Currently, limited evidence exists on the association between nutrition and overtraining syndrome [28]. Prolonged honey consumption can mitigate exercise‐induced immune disruptions in muscles [29]. There are very few studies regarding honey supplementation in military personnel. Mohammed Ali et al. reported that honey increases physical performance at moderate exercise training and decreases the production of inflammatory cytokines and fatigue biomarkers after intense exercise [30]. Despite the differences in subjects and methods, evidence supports the preventive and therapeutic effects of honey on inflammatory cytokines and biomarkers [9, 31, 32]. Experimenting rats with oral or intraperitoneal honey was associated with reduced pro‐inflammatory cytokines such as TNF‐α, IL‐6, NO, COX‐2, PGE2, and CRP highlighting the need for designing human trials [14, 31, 33].

The inflammatory pathway significantly contributes to overtraining syndrome with macrophages playing a pivotal role in muscular regeneration [26, 34]. Intense exercise without sufficient recovery can traumatize the musculoskeletal components, and subsequently release pro‐inflammatory cytokines such as TNF‐α which is implicated in overtraining [35, 36]. The results of our research support the cytokine hypothesis, showing increased TNF‐α levels post‐overtraining, with the intervention group exhibiting a significantly lower increase in serum TNF‐α levels compared to controls. Similarly, a study on healthy male cyclists reported that after 8 weeks of intensive training, raised TNF‐α levels, though honey supplementation mitigated this increase [37]. Conversely, a randomized control trial on inflammatory markers in chronic smokers reported a significant increase in TNF‐α levels with honey supplementation [38]. In another study, no significant TNF‐ α changes were observed in a group of 10 participants aged 20‐30 years undergoing high‐intensity training along with honey supplementation [16]. Additionally, an animal study demonstrated that Saudi honey reduced TNF‐α in indomethacin‐induced gastric ulcers [39]. While our study focused on participants with a normal BMI, Hadiono et al., found no significant impact of high‐intensity training on TNF‐a levels in obese subjects [40].

Despite the limited evidence on correlations between CRP levels and overtraining syndrome [41], some suggest it as a biochemical marker of overtraining [35]. A study by Cadegiani et al. on biochemical predictors of overtraining syndrome failed to indicate a meaningful difference in CRP levels between healthy and overtrained athletes [42]. However, our study observed increased CRP levels in both intervention and control groups after 6 weeks of intensive exercise with a more significant increase in the controls. Supporting these findings, a study by Ghazali et al., showed notably lower post‐intervention levels of high‐sensitive CRP (Hs‐CRP) in chronic smokers after 12 weeks of honey supplementation [38]. Similarly, a clinical trial by Yaghoobi et al. found that natural honey intake led to a significant decrease in Hs‐CRP levels by 3.2% among participants with normal and by 3.3% in those with elevated variables, along with reduced cardiovascular risk factors [43]. Sadeghi et al.'s trial on type‐2 diabetes patients demonstrated a significant decrease in Hs‐CRP concentrations after 8 weeks of honey supplementation combined with dietary recommendations [44]. Another study reported a 7% reduction in CRP levels in healthy individuals and a 57% reduction in hyperlipidemic individuals after 15 days of natural honey consumption [7].

Aldolase A may be used as a biomarker in myopathies and exercise‐induced muscle injuries [45]. Due to the high content of aldolase isoenzyme‐A in muscles, resting serum aldolase activity is considerably greater in athletes after intensive training compared to the nonathlete population [46, 47]. The serum levels of aldolase A were elevated in both groups of our study, which aligns with prior studies. This increment was significantly lower in those who received honey supplementation. However, our exploration of medical literature yielded no studies investigating any link between honey supplementation and serum aldolase levels.

Muscle proteins, such as creatine kinase, are useful biomarkers for assessing muscle damage after intense exercise [48]. A cohort study on professional volleyball players over 16 weeks of training showed an initial surge in CK levels, followed by maintenance during the exercise period and a final decrease [49]. Similarly, in our study, serum CK levels increased in both groups compared to their baselines, but post‐intervention levels were significantly lower in the intervention group than in the controls. Supporting our findings, 11 trained male rowers who consumed 150 mL of a honey solution immediately before and every 15 min during exercise experienced a smaller increase in CK levels (46 U/L) compared to the control group (322 U/L) [50]. Another study found that daily honey consumption over 2 weeks reduced CK levels by one‐third in 10 healthy participants [51]. Additionally, honey supplementation in basketball players' physical recovery was shown to reduce serum CK concentration [52]. Conversely, the survey by Aly et al. on 50 runners reported no significant difference in creatine kinase‐muscle/brain (CK‐MB) levels between the interventions and placebo groups [53].

The present study focused on a specific population of military graduates undergoing overtraining, it does present limitations regarding the generalizability of the findings. However, according to the world population reviews, most nations in the world like Iran, South Korea, and Russia have some form of mandatory military services and share similar military training frameworks. In these nations, a significant portion of the population, particularly young males, undergoes mandatory training, which can lead to overtraining‐related issues [54]. Our findings on the benefits of honey supplementation in reducing inflammation and enhancing recovery could be generalized to other conscripted military personnel across the world, highlighting the broader significance and potential impact of our research.

### 4.1 Recommendations

Considering the widespread practice of mandatory military service in many countries and the associated risk of overtraining, future research should aim to expand on the findings of this study by including a larger and more diverse sample of military personnel. Longitudinal studies should be conducted to evaluate the long‐term effects of honey supplementation on muscle inflammation and recovery. It is recommended to measure a wider array of inflammatory and metabolic markers, such as interleukin‐6 (IL‐6) and interleukin‐10 (IL‐10) at the same time and during long‐term follow‐up, to provide a more comprehensive understanding of changes in gene expression, protein expression, intracellular signaling pathways, and metabolite formation in response to specific chemicals of honey. Additionally, advanced monitoring techniques, such as wearable devices, should be considered to objectively track physical performance related parameters, physiological responses, compliance with supplementation protocols and body compositions. This approach would enhance the accuracy of the findings and help develop evidence‐based guidelines for managing overtraining and nutritional plans in military settings.

### 4.2 Limitations

Despite the noteworthy findings, this study has several limitations. First, the relatively small sample size of military graduates may limit the generalizability of the results to broader populations. Second, the study duration of 6 weeks might not capture the long‐term effects of honey supplementation on muscle inflammation and recovery, necessitating longer follow‐up studies. Third, the investigation was constrained to specific inflammatory biomarkers due to funding limitations, leaving out other potential markers that could provide a more comprehensive understanding of the inflammatory response. Although participants were selected from a specialized military unit with comparable anthropometric profiles, the physical status and body composition were not assessed limiting the evaluation of the intervention's functional impact beyond biochemical markers. Additionally, we did not measure variables such as blood sugar levels, which is particularly relevant to honey consumption.

## 5 Conclusions

This study demonstrates that honey supplementation significantly reduces inflammatory biomarkers such as TNF‐α, CRP, aldolase A, and CK in military graduates experiencing overtraining, highlighting its potential as a natural intervention for managing inflammation related to intense physical training. These findings support the inclusion of honey in dietary strategies aimed at reducing overtraining‐related injuries. To confirm these results and explore their applicability in broader populations, further research with larger sample sizes and extended follow‐up are recommended. Additionally, incorporating a broader range of inflammatory and metabolic markers, as well as detailed evaluations of body composition and physical performance, would provide a more comprehensive understanding of the intervention's effects.

## Author Contributions

**Esmail Karami:** conceptualization, data curation, formal analysis, funding acquisition, investigation, methodology, project administration, resources, software, validation, visualization, supervision. **Mohammad Reza Parvizi:** conceptualization, data curation, formal analysis, investigation, methodology, project administration, resources, software, validation, visualization, supervision. **Mohammad Reza Izadi:** investigation, methodology, supervision, validation, visualization, writing–review and editing. **Emad Jowhari Shirazi:** data curation, formal analysis, funding acquisition, investigation, visualization, writing–original draft, writing–review and editing.

## Conflicts of Interest

All authors contributed to the writing and reviewing of this paper. No conflicts of interest are detected.

## Transparency Statement

The lead author Esmail Karami, Emad Jowhari Shirazi affirms that this manuscript is an honest, accurate, and transparent account of the study being reported; that no important aspects of the study have been omitted; and that any discrepancies from the study as planned (and, if relevant, registered) have been explained.

## Data Availability

SPSS data of the participants can be requested from the authors. Please write to the corresponding author if you are interested in such data.
